# A Multi-Feature and Multi-Level Matching Algorithm Using Aerial Image and AIS for Vessel Identification

**DOI:** 10.3390/s19061317

**Published:** 2019-03-15

**Authors:** Supu Xiu, Yuanqiao Wen, Haiwen Yuan, Changshi Xiao, Wenqiang Zhan, Xiong Zou, Chunhui Zhou, Sayed Chhattan Shah

**Affiliations:** 1 School of Navigation, Wuhan University of Technology, Wuhan 430063, China; sp_xiu@whut.edu.cn (S.X.); hw_yuan@whut.edu.cn (H.Y.); cs_xiao@hotmail.com (C.X.); zwq626197298@whut.edu.cn (W.Z.); zx2000@whut.edu.cn (X.Z.); church_zhou@whut.edu.cn (C.Z.); 2 National Engineering Research Center for Water Transport Safety, Wuhan 430063, China; 3 Intelligent Transportation Systems Research Center, Wuhan 430063, China; 4 Hubei Key Laboratory of Inland Shipping Technology, Wuhan 430063, China; 5 Department of Information and Communication Engineering, Hankuk University of Foreign Studies, Seoul 02450, Korea; shah@hufs.ac.kr

**Keywords:** Unmanned Aerial Vehicle (UAV), vision, Automatic Identification System (AIS), vessel identification, maritime monitoring

## Abstract

In order to monitor and manage vessels in channels effectively, identification and tracking are very necessary. This work developed a maritime unmanned aerial vehicle (Mar-UAV) system equipped with a high-resolution camera and an Automatic Identification System (AIS). A multi-feature and multi-level matching algorithm using the spatiotemporal characteristics of aerial images and AIS information was proposed to detect and identify field vessels. Specifically, multi-feature information, including position, scale, heading, speed, etc., are used to match between real-time image and AIS message. Additionally, the matching algorithm is divided into two levels, point matching and trajectory matching, for the accurate identification of surface vessels. Through such a matching algorithm, the Mar-UAV system is able to automatically identify the vessel’s vision, which improves the autonomy of the UAV in maritime tasks. The multi-feature and multi-level matching algorithm has been employed for the developed Mar-UAV system, and some field experiments have been implemented in the Yangzi River. The results indicated that the proposed matching algorithm and the Mar-UAV system are very significant for achieving autonomous maritime supervision.

## 1. Introduction

As we know, UAV technology has been developed to be more independent and intelligent. Unmanned aerial vehicles (UAVs) have low cost, good flexibility, low risk and high efficiency. Therefore, UAVs have been widely used in various tasks, such as modern maritime supervision [1,2], information collecting [3,4], search and rescue [5,6], environmental monitoring [7,8], exploration and mapping [9,10], etc. 

In typical maritime supervision, live monitoring from the UAV carrying a camera not only provides a broad and steady view but also has excellent mobility. In general, only vessel detection could be achieved depending on the onboard vision. However, more information such as load, goods, power system, etc., need to be acquired for maritime supervision. For example, the vessels carrying dangerous chemicals should be approached by a more careful operation. The AIS (Automatic Identification System) of each vessel can provide the information by broadcast. As a result, combining vision and AIS can achieve identification and tracking. Aniceto et al. [11] presented a study based on field trials using UAVs to carry out the image-based monitoring of cetaceans in two fjords in northern Norway. Comba et al. [12] proposed using the unmanned aerial vehicle (UAV) multi-spectral generated 3D point cloud image to accurately detect vineyards, which plays a vital crop monitoring function in the viticulture process. Ribeiro et al. [13] presented a dataset with surveillance imagery over the sea that was captured by a small size UAV. This dataset presents object examples ranging from cargo ships, small boats, life rafts to hydrocarbon slick. Zhang et al. [14] used a low-altitude unmanned aerial vehicle (UAV) remote-sensing platform equipped with an optical digital camera to inspect power line corridors. Freitas et al. [15] addressed the use of a hyperspectral image system to detect vessels in maritime operational scenarios. The developed hyperspectral imaging classification methods are based on supervised approaches and allow for the detection of the presence of vessels using real hyperspectral data. We implemented two different methods for comparison purposes: SVM (Support Vector Machine) and SAM (Spectral Angle Mapper).

The real-time images of vessels and monitoring environment are captured sequentially by a camera mounted on the UAV. The detection or tracking could be achieved through some image segmentation and matching techniques. However, these detected vessels cannot be identified using only because their real names or IDs are unknown. It is not beneficial to maritime law enforcement. Alternatively, the vessel information involving name, current heading, speed and other attributes can be obtained from an onboard AIS receiver. Through the collaboration of vision and AIS, the vessels could be detected and identified. 

In recent years, there has been a wealth of research and various vision-based methods made available for Maritime UAV applications. The related research includes the design and control of UAV research [16], business application mode and method research [17], task-oriented path planning and collaborative operation research [18,19]. Ross et al. [20] introduced a ship detection and identification system based on the fusion of multiple sensors (including vessel AIS equipment, satellite imagery and radar images). Habtemariam et al. [21] proposed a radar and AIS information fusion algorithm based on the joint probability data protocol framework. The uncertainty of the AIS identification number is solved by assigning multiple AIS identification numbers to the target and updating the identification number probability according to Bayesian inference. This algorithm combines radar and AIS information.

Lang et al. [22] proposed an adaptive multi-sample transfer learning method by combining Synthetic Aperture Radar (SAR) and AIS for ship detection and tracking. There is insufficient data in existing ship classification samples in SAR images, but the classification of ships in the AIS information is clear with sample-rich data. SAR is a high-resolution microwave imaging radar using the principle of synthetic aperture. The AIS dataset is used to train the SAR image, which reduces the ship detection and classification error rates. Pelich et al. [23] and Zhao et al. [24] selected the minimum distance matching between the feature information such as the position, heading and ship length and the AIS information whilst considering the incident angle, polarization, frequency and spatial resolution of the SAR sensor. In this way, the false alarm rate is reduced, improving ship detection and identification.

Satellite imagery, radar imagery, ship AIS data detection and identification are mature compared to aerial imagery and ship AIS data detection and recognition. It is an important research direction to improve the autonomy of UAVs in current maritime supervision applications, especially for realizing the automatic recognition and tracking of targets based on unmanned aerial sensors (such as cameras and laser radars) [25]. Currently, UAVs use onboard vision to capture maritime scenes and send real-time images to the ground station. All further processing, especially vessel identification, has to be done manually by the staff. Once the matching between image-based detection and the AIS message is confirmed, vessel identification could be achieved automatically by UAVs. In this paper, a Mar-UAV system was developed by a large-scale multi-rotor aerial vehicle equipped with a high-resolution camera and a customized AIS receiver. For the purpose of autonomous vessel identification, a multi-featured and multi-level matching algorithm was proposed to match the image-based vessel detection with the AIS message. Except for the features including position, vision-based localization, heading, speed, etc., a hierarchical structure using point to trajectory matching has been considered in the algorithm. Depending on such a matching algorithm, the Mar-UAV system has the ability to detect and identify vessels automatically instead of manually. In the end, by using the Mar-UAV system, some field experiments were implemented over the Yangzi River. The results were also displayed to illustrate the proposed algorithm and the Mar-UAV system. The proposed matching algorithm is beneficial in improving the autonomy of UAVs in maritime supervision.

The rest of the work is organized as follows. Section 2 introduces the design and development of the Mar-UAV system. Section 3 describes the proposed multi-featured and multi-level matching algorithm. Section 4 presents the experimental results and the performance analysis of vessel detection and identification. Finally, the conclusion is given in Section 5.

## 2. Systematic Design

**Figure 1:**
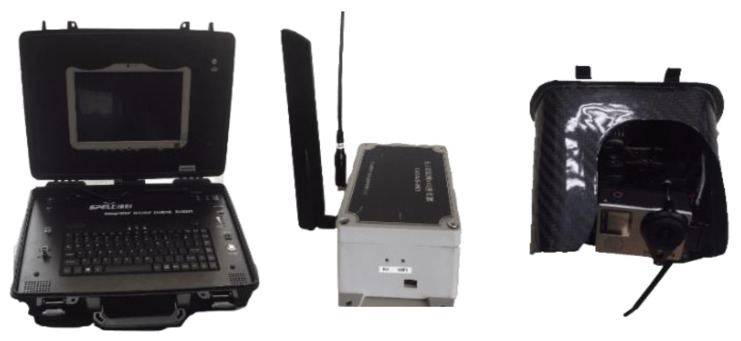
The ground control system, onboard Automatic Identification System (AIS) transceiver and imagery device.

**Figure 2:**
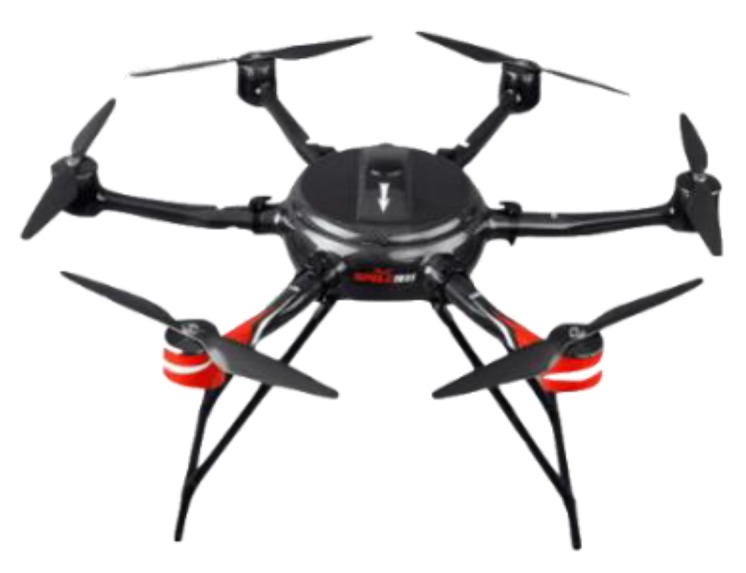
The overall view of the maritime unmanned aerial vehicle (Mar-UAV).

The developed Mar-UAV (maritime unmanned aerial vehicle) system is based on a multi-rotor copter platform. With a 2 Degree-Of-Freedom (DOF) camera mount, a high-resolution camera and an AIS transceiver as the main payload, the Mar-UAV can fly over the river, acquiring videos of the water target. The real-time vision and AIS data are transmitted wirelessly to the ground control system, in which all the algorithms are processed. Additionally, the ground system is responsible for the visualization and supervision of all the acquired data. The multi-featured and multi-level matching algorithm proposed in Section 3.2 can be used to detect and identify the vessels in the view. The Mar-UAV can be operated in a semiautonomous mode or a fully autonomous mode, depending on the field and the specified task. The semiautonomous mode is suitable for searching in a specified short-range region, which can be improved with human supervision. The superior flight maneuverability of the Mar-UAV makes it the most suitable platform for low-altitude remote sensing and evaluation tasks. Figure 1 and Figure 2 show the overall system.

### 2.1. System Architecture

The goal of the Mar-UAV system in this work is to find a target’s GPS coordinates and identify the target, which requires a suitable type of aircraft frame. The aircraft needs enough fuselage space to accommodate the necessary payload for the task. The vehicle configuration and material exhibit good aerodynamic performance and a reliable structural strength for the missions. The propulsion system for the aircraft is calculated once the Mar-UAV’s configuration and requirements are known.

**Figure 3:**
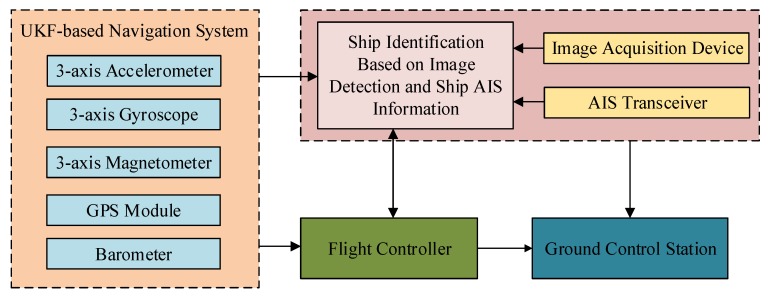
The hardware architecture of the Mar-UAV.

Next, a communication system, including a telemetry system, is used to connect the ground station to the Mar-UAV. After adding the flight control system, the aircraft takes off, following the designed route autonomously. Finally, with the help of the mission system, targets’ and their GPS coordinates can be found. Figure 3 shows the systematic framework of the Mar-UAV system, with the details in the following sub-sections. The whole system weighs 9.45 kg and takes off via hand launching. In order to resist the wind over the Yangzi River, the Mar-UAV system was designed with a strong airframe and a powerful dynamic. Therefore, except for its airframe (1.25 kg), six brushless motors (0.45 kg × 6) and two LiPo battery (2 kg × 2) take up most of the total weight.

### 2.2. Airframe

The Mar-UAV system is established to satisfy the diverse demands in real maritime supervision applications. The iNavA6-100, designed for surveillance, can be used for locating and target recognition with a camera and AIS transceiver as the main payload. The iNavA6-100 is the name of the Mar-UAV system. We developed the system by integrating a six-rotor drone with a high-resolution motion camera and an AIS sensor. The main body of the fuselage adopts high-intensity, high-rigidity imported carbon fiber composite materials and advanced one-piece molding technology. According to the force analysis of the different parts of the fuselage, different processing techniques are used to achieve the lightest weight while ensuring rigidity and strength. The rain-proof design of the entire fuselage is designed to fly in moderate weather. Moreover, the small scale and lightweight design of the iNavA6-100, convenient for maritime supervision, can conduct searches near the accident region. Table 1 shows the Specific parameters of the employed drone.

### 2.3. Propulsion and Navigation

The UAV uses a KWT-8108/6S motor. With a 20,000 mAh, Lipo 6-cell, 15 C battery, this propulsion system provides a maximum cruise time of 40 min at an airspeed of 12 m/s.

The navigation system consists of a three-axis accelerometer, a three-axis gyroscope, a three-axis magnetometer, a GPS module, and a barometer [26], which are integrated into a coupled INS/GPS navigation system [27]. In order to ensure the safety of flight, a differential GPS dual antenna is used for aircraft orientation. This is not affected by the electromagnetic environment as long as the GPS signal is found for the flight positioning. The Mar-UAV with this navigation system can conduct a fully autonomous mission, including auto take-off, cruising via waypoints, returning to its home position and auto landing, with enhanced fail-safe protection.

### 2.4. Ground Communication System

The Ground Communication System (GCS) is a wireless digital radio that can acquire onboard information involving aerial image, AIS, etc., and enables staff to monitor the health state of the Mar-UAV system in real time. The SPELL-IG is an algorithm software integrated into the ground system. This software is mainly responsible for trajectory planning and visualization of the Mar-UAV system. The 900 M-frequency digital transmission station and the 595 M-frequency, 8 M-bandwidth image transmission station are integrated into the GCS. The Mar-UAV is controlled by the GCS for over-the-horizon flight. The maximum control distance is up to 10 km. An auto antenna tracker works in conjunction with a Yagi antenna to provide a reliable data link within a 10-km range. The Yagi antenna is a directional type antenna and can be used for point to point or point to multi-point WiFi applications. The Yagi antenna is responsible for data transmission between the ground system and the Mar-UAV system. The Mar-UAV can not only follow the flight route set in advance in the GCS software, but also modify the route in the GCS software during the flight to achieve autonomous flight.

The AIS transceivers are installed on Mar-UAV to receive AIS messages from surface vessels, which is uploaded to the network server through a 4G communication module. The ground terminal accesses the server through the Internet to obtain and analyze the AIS data.

### 2.5. Post-Imaging Processing and Video Transmission 

GoPro HERO 4 (GoPro, Inc., San Mateo, CA, USA) is a motion camera that can provide high-quality images for our matching algorithm. In our work, the camera is our vision module (see Figure 1), which is installed under the body of the Mar-UAV system. In a searching and mapping mission, the aerial image always faces the ground. During the flight, some actions such as rolling, pitching or other unexpected vibrations can disrupt the camera’s stability, which may lead to an unclear video. A Mini 2D camera gimbal, produced by Keweitai Tech Co., Ltd. (Shenzhen, China) and powered by two brushless motors, was used to stabilize the camera. The camera was set to video mode with a 1920 × 1080 pixel resolution and a width field of view (FOV) at 30 frames per second [28]. During the flight, a digital image signal is sent to an on-screen display and video transmitter. With a frequency of 595 MHz, the aerial video can be visualized by GCS in real time as the high-resolution video is rerecorded for use during post-processing.

## 3. Matching Algorithms, Image, and AIS Data

**Figure 4:**
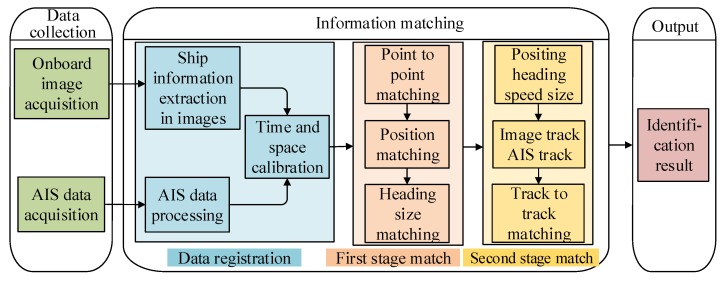
The matching model of the onboard image and AIS information.

Figure 4 shows the framework of the multi-feature, multi-level matching model algorithm for the onboard image and AIS information of the Mar-UAV. It consists of the data acquisition, information matching and output phases. In the data acquisition phase, the onboard camera and the AIS equipment acquire the image information and AIS information of the vessel, respectively. After extracting the target image and preprocessing the AIS information, space-time calibration is performed. This information is used as an input to match the onboard image to the AIS data.

In order to improve the speed and accuracy of the matching, the matching algorithm in the work is divided into two stages. The first stage performs point matching for the position information, heading information and size information of the point to improve the speed of the target recognition. It gradually enters the field of view of the camera. In the second stage, spatial matching is performed by using the trajectory information in the image and the trajectory information of the AIS data in order to improve the target recognition. The identified output can be used as a result of target verification and target tracking.

### 3.1. Image and AIS Information Processing

#### 3.1.1. Image-Based Detection and Localization

(1) Image correction

According to the camera imaging principle, the wide-angle lens has a large field of view, which can quickly capture water targets. However, the image taken by the wide-angle lens has a large distortion. Before detecting the target, the distorted images need to be corrected [29].

First, the camera parameters need to be calibrated. The traditional checkerboard method is used to calibrate the camera. The calibration result is as follows: Internal reference matrix $A=\left[\begin{array}{cc} \begin{array}{cc} \begin{array}{c} 413.68\\ 0 \end{array} & \begin{array}{c} 0\\ 414.82 \end{array} \end{array} & \begin{array}{c} 0\\ 0 \end{array}\\ \begin{array}{cc} 959.72 & 530.70 \end{array} & 1 \end{array}\right]$; Rotation matrix $R=\left[\begin{array}{cc} 0.0425 & -0.0073 \end{array}\right]$; Translation matrix $T=\left[-\begin{array}{cc} 0.0103 & 0.0008 \end{array}\right]$.

**Figure 5:**
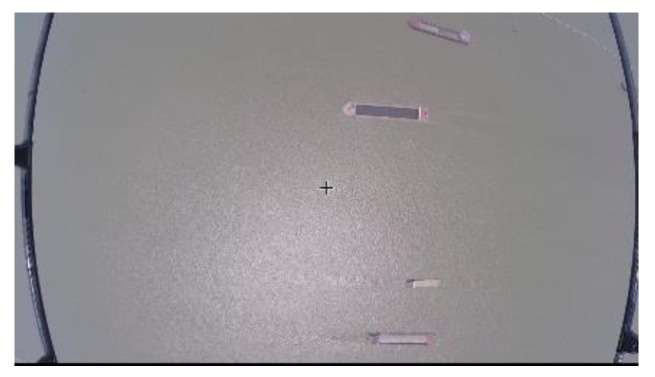
The Original Image.

**Figure 6:**
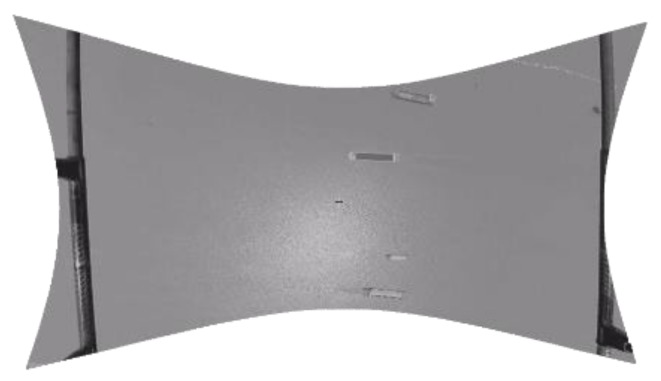
The Rectified Image.

Secondly, the image is rectified by a regression algorithm based on SVM correction. Figure 5 and Figure 6 are shown as original and rectified images, respectively.

(2) Vessel detection

**Figure 7:**
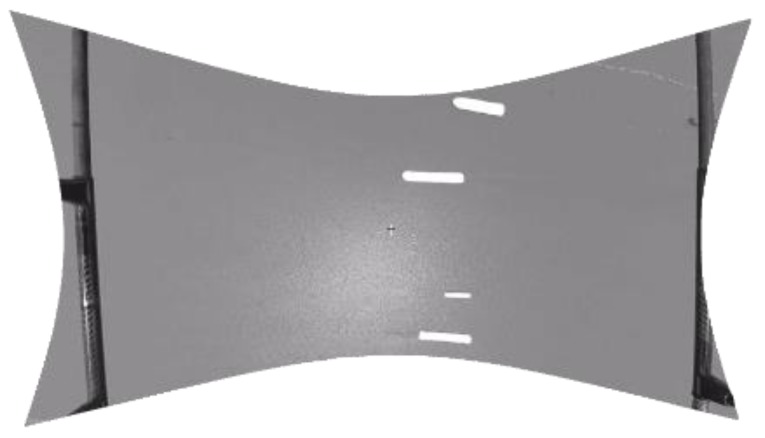
The target detection of the vessel.

The target detection uses the segmentation method based on the structure chart classification to realize vessel detection in the image. Figure 7 shows the detection results. The algorithm includes the following steps: 

Firstly, the original image is converted to a structure diagram (*G*=(V,E)). In this structure, each element represents a vertex in the graph ($V_{i}\in V$) and adjacent vertices form an edge ($\left(V_{i},V_{j}\right)\in E$). The difference in the corresponding grayscale, coordinate, and texture information among the elements constitutes the weight $\omega \left(v_{i},v_{j}\right)$ of the edge $\left(v_{i},v_{j}\right)$. The smaller the value about ω, the higher the similarity between elements.

Then, the graph *G* is reduced to a minimum spanning tree. That is, all elements with high similarity or pixel regions are merged. Each region *C* contains several vertices connected by the edges of the smallest spanning tree. Further, the structure map is merged according to the differences between the regions and similar regions form a branch. The difference between the node regions is formed by internal differences and inter-region differences, while the internal differences refer to the weights of the largest edges in the region *C* (See Equation (1)).

(1) $$Int\left(C\right)=\underset{e\in MST\left(C,E\right)}{\max}\omega \left(e\right)$$

The difference between the regions refers to the weight of the smallest edge where the vertices between the two divided regions are connected to each other. Equation (2) shows the specific calculation.

(2) $$Dif\left(C_{1},C_{2}\right)=\underset{v_{i\in }C_{1},v_{j\in }C_{2},\left(v_{i},v_{j}\right)\in E}{\min}\omega \left(\left(v_{i},v_{j}\right)\right)$$

Finally, the separation or detection of the vessel target in the image is achieved based on the above-mentioned intra-region difference value Int and the difference value *Dif* between the regions. Specifically, a threshold function *τ* is introduced to determine the two values and it is determined whether the detection area contains target information with complicated structure or edges. If there is an obvious edge or contour information, it is thought to be a vessel.

(3) Image-based vessel localization

**Figure 8:**
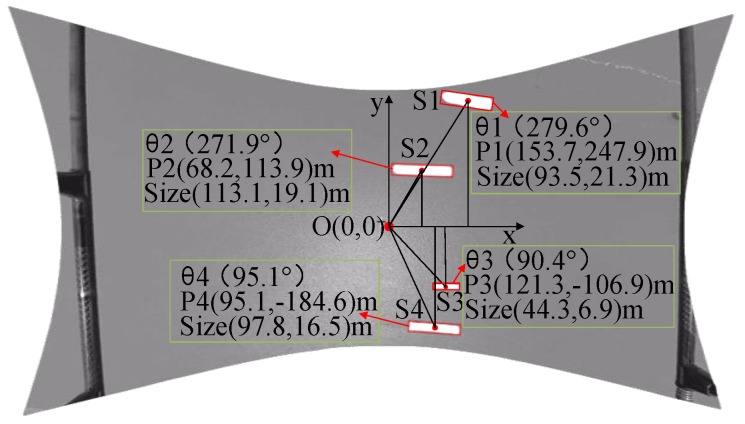
The vessel information extraction.

As shown in Figure 8, four vessels are detected, and their characteristics include course, position and size. Their speeds can also be calculated based on the position of two consecutive frames of pictures.

#### 3.1.2. Processing AIS Information

**Figure 9:**
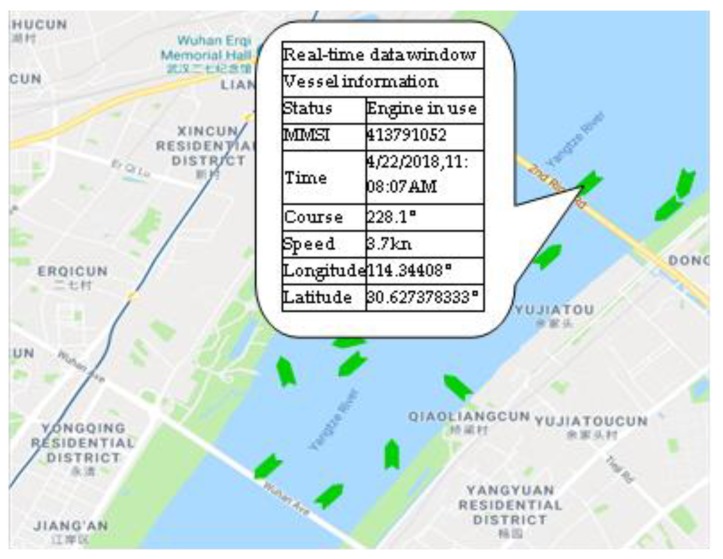
The AIS dynamic information displayed on the map.

According to the algorithm, the AIS information is analyzed to extract the MMSI, name, location, speed, heading and size information of the vessel. The effective dynamic information includes MMSI, latitude and longitude, ground speed and ground heading (See Table 2); the effective static information includes name, MMSI, vessel length and width [30] (See Table 3). Figure 9 shows a vessel’s dynamic information received in real time on the map.

#### 3.1.3. Calibration of the Image and AIS Information

(1) Space calibration

Since the onboard image is positioned in the image coordinate system, with the positioning of the AIS information in the Earth’s coordinate system, it needs to be converted to a unified coordinate system for data matching through spatial calibration. The work proposes an onboard image localization algorithm based on the Earth’s coordinate system, which locates the target under the following three assumptions:

(1) Since the GPS module is located above the onboard camera, the Mar-UAV is at the center of the image.
(2) Since the onboard camera is mounted on the pan-and-tilt, the camera’s shooting angle should be set perpendicularly to the ground.
(3) ψ is the angular deviation for the transformation of a north-east (NE), world-to-camera frame.

**Figure 10:**
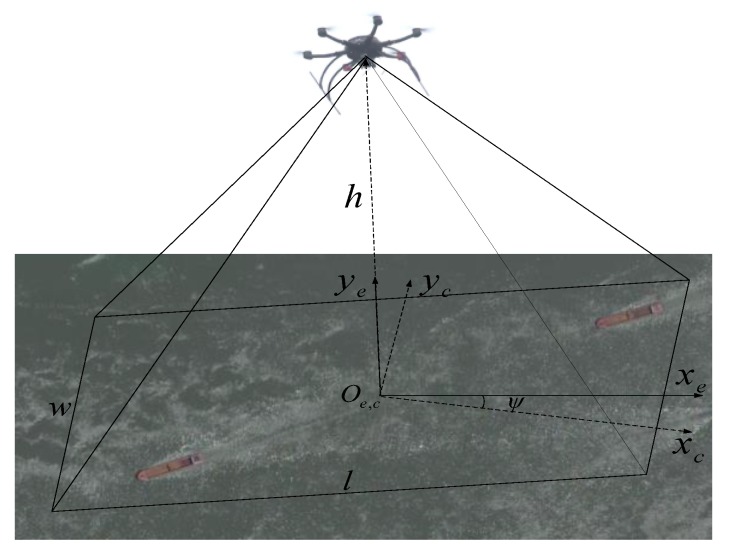
The camera and earth coordinates.

The range of the field of view coverage can be estimated based on the camera’s field of view, the UAV’ height, and GPS position information (see Figure 10). The field of view can be calculated by Equation (3).

(3) $$\{\begin{array}{c} w=\frac{2h}{\cos\left(\theta_{x}/2\right)}\\ l=\frac{2h}{\cos\left(\theta_{y}/2\right)} \end{array}$$

where w and l denote the distances of the field of view length and width, respectively; $\theta_{x}$ and $\theta_{y}$ denote the camera angles of view on the x- and y-axes, respectively.

The resolution of the video frame is set to 1920 × 1080 pixels. The scale between the distance and pixels is assumed to be in a linear relation vessel (see Equation (4)).

(4) $$\{\begin{array}{c} pixel_{x}=\frac{w}{1920}=\frac{2h}{1920\cdot \cos\left(\theta_{x}/2\right)}\\ pixel_{y}=\frac{l}{1080}=\frac{2h}{1080\cdot \cos\left(\theta_{y}/2\right)} \end{array}$$

The target is assumed to be located on the (*x*, *y*) pixel in the photo, and the offset of the target from the center of the image is

(5) $$offset_{target}=\left[\begin{array}{c} pixel_{x}\cdot x\\ pixel_{y}\cdot y \end{array}\right]$$

The conversion matrix of the camera coordinate system $O_{c}$ to the earth coordinate system $O_{e}$ is

(6) $$R_{c}^{e}=\left[\begin{array}{cc} \cos\left(\psi \right) & -\sin\left(\psi \right)\\ \sin\left(\psi \right) & \cos\left(\psi \right) \end{array}\right]$$

The position offset in the earth frame can be solved with

(7) $$P=R_{c}^{e}offset_{target}=\left[\begin{array}{c} P_{E}\\ P_{N} \end{array}\right]$$

The target’s GPS coordinates can be determined by

(8) $$GPS_{target}=GPS_{cam}+\left[\begin{array}{c} P_{E}/f_{x}\\ P_{N}/f_{y} \end{array}\right]$$

where $f_{x}$ and $f_{y}$ denote the distances of one degree of longitude and latitude, respectively.

(2) Time calibration

Since the onboard image and AIS are two separate sensors, the collected data is stored separately. Therefore, spatial matching needs time calibration, including time-based calibration and sampling-period calibration.

(1) A pulse signal is generated by the hardware to start the onboard camera and the AIS device, thus, ensuring the synchronization of the sampled data head.
(2) Synchronization of the sampling period. The sampling period of the image is 33 ms, and the receiving period of the AIS data is 2–180 s. So it is necessary to synchronize the two different data. Considering that the sensors with different data frequencies need to be time aligned, we employed simplified filtering to interpolate some estimation values between the data of the AIS receiver with a lower frequency. The filtering is based on a linear kinematic model. This assumption is reasonable because the motion of a vessel is thought to be constant for short time periods. In detail, the filtering linearly interpolates the AIS data to 1 Hz and samples the image information to 1 Hz, thus ensuring the synchronization of the data sampling period.

### 3.2. Multi-Featured and Multi-Level Matching Algorithm

#### 3.2.1. Multiple Feature Selection

According to the principle of image detection, the characteristics of the vessel extracted from the airborne image include size, location, head and grayscale statistics. The AIS information includes the vessel’s MMSI, name, location, size, ground heading and ground speed. By comparing their characteristics, the matched position, size and heading can be obtained.

(1) Location feature

The position information of vessels, obtained by the positioning algorithm of the target and by the AIS information, should be matched.

(2) Geometric features

The length and width of a vessel calculated by AIS are obtained by calculating the distance from the information transmission point to the bow, stern, left chord and right chord. In order to improve the matching accuracy, the appropriate reference point is selected with the extracted length and width of the vessel. Moreover, the ratio of the length and the width of the vessel is used for matching. The calculation of the heading is based on the angle between the main axis of the vessel and the true north direction.

(3) Movement characteristics

The relative position of a vessel can be obtained by detecting the vessel between two consecutive images. The speed of the vessel can be calculated by combining the acquisition time interval of the image. This speed can be matched to the speed of the vessel analyzed by the AIS.

#### 3.2.2. Multi-Level Hierarchical Matching

The UAV fixed-point hovering method is used to quickly and accurately identify vessels entering the perspective of the airborne camera. Since the vessel gradually enters the field of view of the camera, target matching is divided into two stages according to the detection purpose—the point matching stage and the track matching stage.

(1) Point to track matching

**Figure 11:**
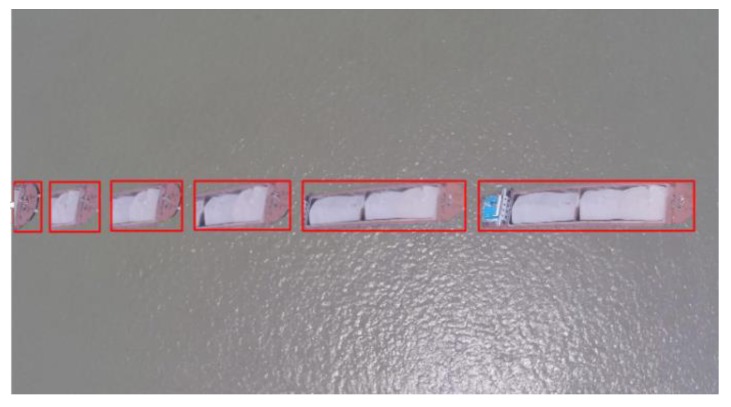
Consecutively detected vessels in a real-time video.

The point matching phase is to quickly match the vessel entering the camera’s field of view. Figure 11 shows the first stage of the match. The vessel is gradually detected in the field of view of the camera. A target point to track matching method is employed in the process. First, the detected target is positioned in the image coordinate system. Then, the target position is calibrated according to the spatial calibration algorithm (converted to the geodetic coordinate system). This position is matched to the acquired AIS information. Finally, the heading and size matching are further carried out on the target with the correct position matching.

(2) Track to track matching

In order to ensure the accuracy of the matching, the second phase of the trajectory matching is performed on the target. First, a period of data acquisition and formation of a target trajectory is performed on a target within the field of view of the camera. Secondly, the tested heading, speed, position and size characteristics are matched to the trajectory formed by the AIS information to improve the matching accuracy.

#### 3.2.3. The Multi-Featured and Multi-Level Matching Algorithm

(1) Point to track matching algorithm

If there are *M* vessels in the image, their positions are $P_{1},P_{2},\ldots ,P_{M}$; if there are *N* vessels in the AIS information, their positions are $Q_{1},Q_{2},\ldots ,Q_{N}$, respectively. The position of the vessel is matched. If the vessel numbered *P* in the image matches the vessel numbered *Q* in the AIS information with the shortest position, the output squared error is the smallest.

(9) $${\left|r\left(p,q\right)\right|}^{2}={\left|P_{p}-Q_{q}\right|}^{2}$$

According to the analysis of the position matching error, the threshold d needs to be set because the vessel AIS data may be false-alarm, or the vessel has no AIS information. When r<d, the position is considered to be correct. Then, the headings and sizes of the two vessels with the correct position matching are matched; if the error is within a certain allowable range, the matching is correct.

(2) Track to track matching algorithm

In the work, the vessel’s trajectory information includes heading, speed, position and length of the trajectory. The matching of the trajectory of the vessel can be converted into a match to the trajectory feature information. That is, we can obtain the correlation and similarity between the vessel’s trajectory of image detection and the AIS trajectory in the feature information.

Structural similarity index (SSIM) can be expressed by Equation (10):

(10) $$M\left(I,A\right)=Dir\times W_{D}+Speed\times W_{S}+Loc\times W_{L}$$

where F=[Dir,Speed,Loc] is the difference quantity of the feature, and $W=\left[W_{D},W_{S},W_{L}\right]$ is the weight of the response feature. In the matching process, the relative difference of the position information is large, so the weight $W_{L}$ is set as a function variable. When the vessel numbered $P_{1}$ in the image matches the vessel numbered $Q_{1},Q_{2},\ldots ,Q_{N}$ in the AIS information, the two minimum values $Loc_{min}$ and $Loc_{min-1}$ are selected in the comparison of position $\left(Loc_{11},Loc_{12},\ldots ,Loc_{1N}\right)$. When the difference $e_{loc}=\left|Loc_{min}-Loc_{min-1}\right|$ is less than *d*, it means that the two vessels are similar in position and the weight of $W_{L}$ needs to be weakened.

(11) $$W_{L}=\{\begin{array}{c} \;\xi \;e_{Loc}\geq d\;\\ 0.2\xi \;e_{Loc}<d \end{array}$$

(1) Heading comparison

(12) $$Dir\left(I,A\right)=\{\begin{array}{cc} \begin{array}{c} \min\left(\left\|I\|,\|A\right\|\right)\times \sin\left(\theta \right),\\ \min\left(\left\|I\|,\|A\right\|\right), \end{array} & \begin{array}{c} \;0\leq \;\theta \leq 90\\ 90\leq \;\theta \leq 180 \end{array} \end{array}$$

where ‖I‖ and ‖A‖ represent the image track length and the AIS track length, respectively; *θ* is the angle between the two tracks.

(2) Speed comparison

(13) $$Speed\left(I,A\right)=\frac{1}{3}\left(S_{max}\left(I,A\right)\right)+S_{avg}\left(I,A\right)+S_{min}\left(I,A\right)$$

where $S_{max}\left(I,A\right)$ is the absolute value of the maximum speed difference between the two tracks; $S_{avg}\left(I,A\right)$ and $S_{min}\left(I,A\right)$ are the absolute values of the average speed and the minimum speed difference, respectively. The speed describes the difference of the overall speed from the maximum speed, minimum speed and average speed.

(3) Position comparison

We calculate the average distance of the vessel’s trajectory detected by the image and the corresponding discrete point on the vessel’s AIS information trajectory.

(14) $$Loc\left(I,A\right)=\frac{1}{n}\sum_{i=0}^{n}\left|D_{Ii}-D_{Ai}\right|$$

where $D_{Ii}$ is the vessel’s position of the discrete point in the image; $D_{Ai}$ the vessel’s position of the AIS information.

#### 3.2.4. Error Analysis of the Matching Algorithm

Position matching refers to the matching between aerial image-based vessel positioning and the position report from the AIS receiver. The errors of the above-mentioned matching algorithm come mainly from image-level detection error $E_{L}$ and AIS error $E_{A}$. For the employed image-based positioning algorithm, the global position of the vessel can be calculated by using the onboard GPS of the Mar-UAV. As a result, the GPS measurement error $E_{g}$ is also considered together with image detection error $E_{Im}$. The two errors $E_{g}$ and $E_{Im}$ are modeled respectively with a Gaussian white noise that the mean is zero and the variance is σ. In addition, the AIS error consists of two parts, measurement error $E_{Am}$ and calculation error $E_{Ac}$. The measurement error $E_{Am}$ is determined by the GPS accuracy of the AIS module. Since the receiving period of the AIS message is uncertain, filtering-based interpolation is necessary. Thus, some calculation error exists in the interpolation processing. The two errors of AIS can also be defined with Gaussian white noises. So the errors of the multi-featured and multi-level matching algorithm introduced in this section can be expressed using the following equation:

(15) $$\{\begin{array}{c} E_{L}=E_{g}\left(0,\sigma_{g}\right)+E_{Im}\left(0,\sigma_{Im}\right)\\ E_{A}=E_{Am}\left(0,\sigma_{Am}\right)+E_{Ac}\left(0,\sigma_{Ac}\right) \end{array}$$

## 4. Experimental Results and Analysis

### 4.1. Point to Track Matching Results and Analysis

Firstly, the absolute position and heading of the vessel in the earth coordinate system are calculated by Equations (4)–(8), according to the height and position of the UAV, the horizontal declination of the camera, the angle of view, and the relative coordinates of the vessel. Then, the point to track matching algorithm is used to match the vessel to obtain the information of the inspected vessel.

**Figure 12:**
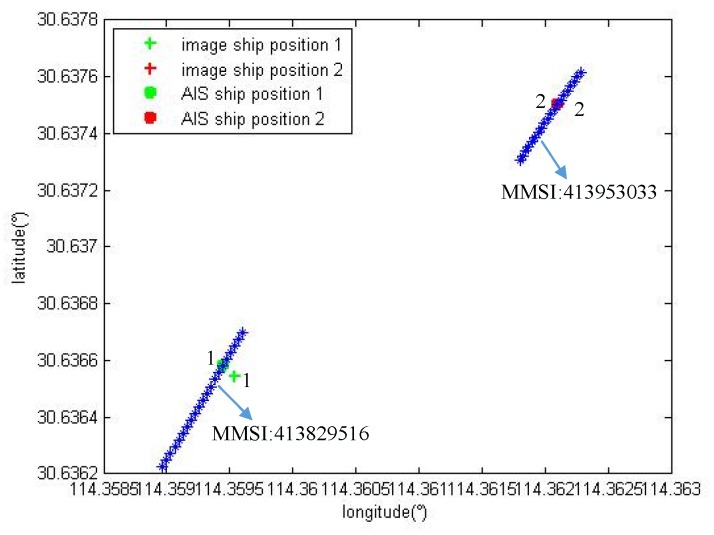
The results of the point to track matching algorithm for the identification of two vessels.

**Figure 13:**
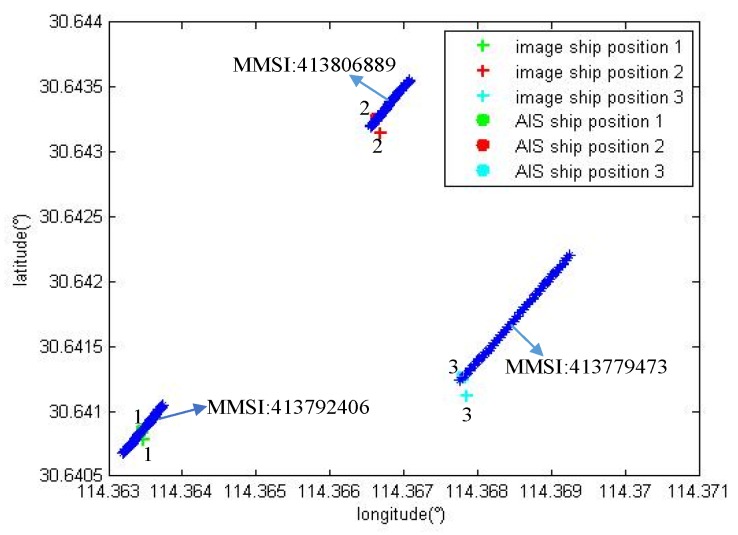
The results of the point to track matching algorithm for the identification of three vessels.

**Figure 14:**
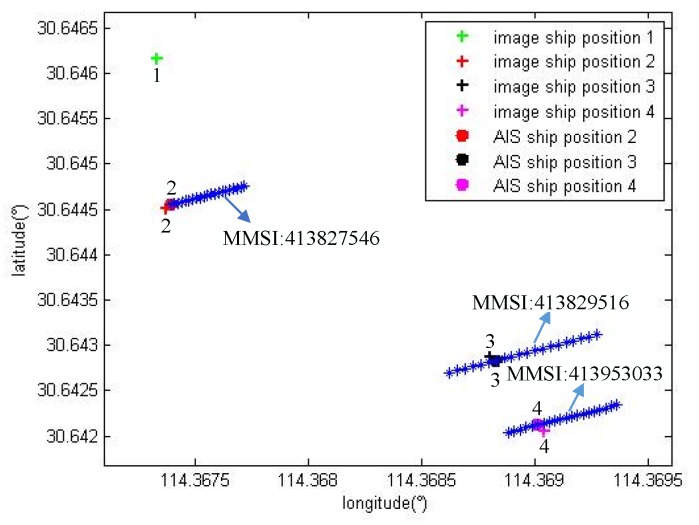
The results of the point to track matching algorithm for the identification of four vessels.

Three sets of experiments were performed using the point to track matching algorithm to match two, three and four vessels in the onboard image. Figure 12, Figure 13 and Figure 14 show the experimental results.

(1) Matching two vessels

The measurement parameters are as follows: height h=205.0 m; position P(lon,lat)=(114.36157227°,30.63745689°); camera horizontal declination ψ=318.3°; threshold d=30 m.

As shown in Figure 12, + indicates the position of the two vessels detected by the image and the blue * indicates the vessel AIS data track. The position matching minimum error $E_{M1}=8.7\;m$, and $E_{M2}=3.5\;m$. The error is less than the threshold *d*. The heading and size matching of the vessel are less than the set threshold.

(2) Matching three vessels

The measurement parameters are as follows: height h=365.2 m; position P(lon,lat)=(114.36662292°,30.64152145°); camera horizontal declination ψ=324.7°; threshold d=30 m.

As shown in Figure 13, + indicates the position of the three vessels detected by the image and the blue * indicates the vessel AIS data track. The position matching minimum error $E_{M1}=7.4\;m$; $E_{M2}=12.2\;m$; $E_{M3}=15.7\;m$. The error is less than the threshold *d*. The heading and size matching of the vessel are all less than the set threshold.

(3) Matching four vessels

The measurement parameters are as follows: height h=360.3 m; position P(lon,lat)=(114.36743164°,30.64311218°); camera horizontal declination ψ=327.9°; threshold d=30 m.

According to the point to track matching algorithm, the position of the vessel is calculated in the image to match the AIS data near the time. As shown in Figure 14, + indicates the position of the four vessels detected by the image and the blue * indicates the vessel AIS data track. There is only the information of three vessels that can be received from the AIS, indicating that there is a vessel without AIS information. The position matching minimum error $E_{M1}=141.4\;m$; $E_{M2}=4.7\;m$; $E_{M3}=4.3\;m$; $E_{M4}=7.4\;m$. The green + indicates that the vessel numbered 1 has no matching vessel in the AIS data because the minimum position *r* is larger than the threshold *d*. Next, the heading and size of the vessel with the correct position match are matched, with the error less than the set threshold.

Therefore, the point to track matching algorithm can match the vessel detected in the image and AIS information. The method can identify the detected vessel.

### 4.2. Track-to-Track Matching Results and Analysis

As the vessel’s time in the field of view of the camera increases, the vessel forms a trajectory detected in the image to match the AIS trajectory. The matching result has higher accuracy than the point to track matching.

According to the track-to-track matching algorithm, if $W_{D}=1$, $W_{S}=1$, and ξ=1, the matching results are *M1*, *M2* and *M3*. Where, $M_{ij}$ represents the structural similarity of the trajectory of the vessel numbered i and the trajectory of the vessel numbered j in the AIS data. A smaller value leads to more similarity. The experimental data are analyzed according to the driving characteristics of the inland river vessel. When the similarity value is less than 50, the two trajectories are correct.

**Figure 15:**
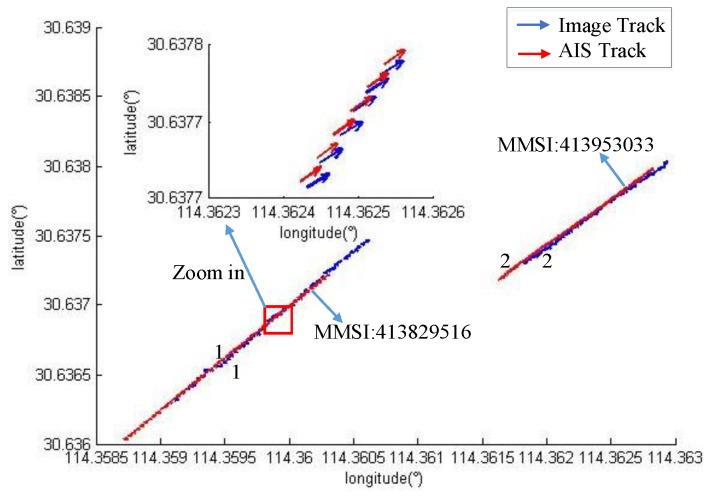
The identification results for two vessels.

**Figure 16:**
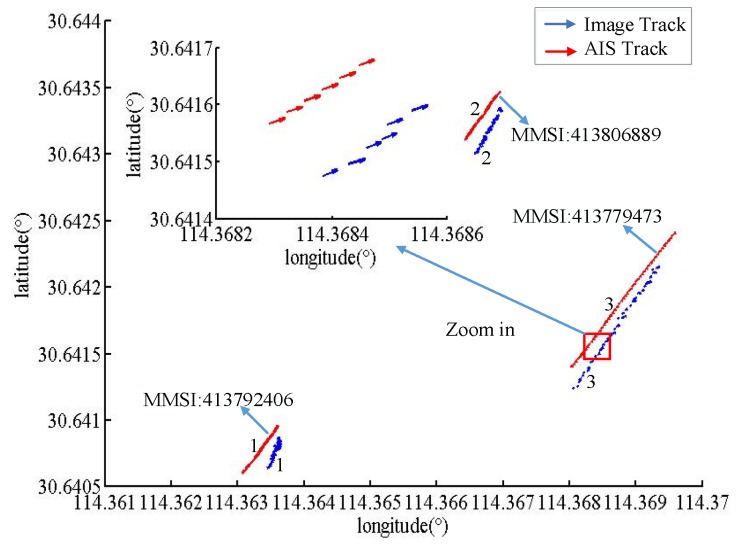
The identification results for three vessels.

**Figure 17:**
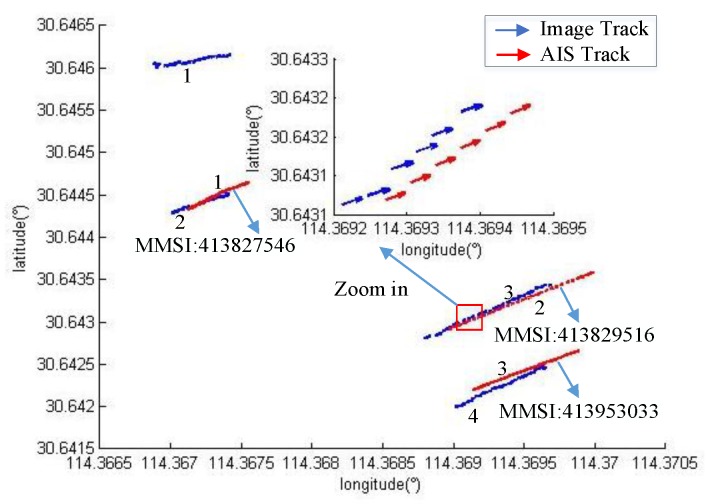
The identification results for four vessels.

Figure 15, Figure 16 and Figure 17 show the vessel’s trajectory matching results. The small picture is a partial enlargement of the large picture, which shows that each discrete point represents the position and heading of the vessel.

$$M1=\left[\begin{array}{cc} M_{11} & M_{12}\\ M_{21} & M_{22} \end{array}\right]=\left[\begin{array}{cc} 37.4281 & 299.8045\\ 337.0242 & 22.1018 \end{array}\right]$$

$$M2=\left[\begin{array}{cc} M_{11} & \begin{array}{cc} M_{12} & M_{13} \end{array}\\ \begin{array}{c} M_{21}\\ M_{31} \end{array} & \begin{array}{c} \begin{array}{cc} M_{22} & M_{23} \end{array}\\ \begin{array}{cc} M_{32} & M_{33} \end{array} \end{array} \end{array}\right]=\left[\begin{array}{ccc} 31.2307 & 429.7690 & 720.0474\\ 449.6429 & 23.0316 & 365.0460\\ 675.3110 & 351.4984 & 26.2097 \end{array}\right]$$

$$M3=\left[\begin{array}{cc} M_{11} & \begin{array}{cc} M_{12} & M_{13} \end{array}\\ \begin{array}{c} M_{21}\\ \begin{array}{c} M_{31}\\ M_{41} \end{array} \end{array} & \begin{array}{c} \begin{array}{cc} M_{22} & M_{23} \end{array}\\ \begin{array}{cc} \begin{array}{c} M_{32}\\ M_{42} \end{array} & \begin{array}{c} M_{33}\\ M_{43} \end{array} \end{array} \end{array} \end{array}\right]=\left[\begin{array}{ccc} 166.6384 & 453.2877 & 514.2250\\ 20.7034 & 335.2119 & 337.2014\\ \begin{array}{c} 310.7837\\ 369.1452 \end{array} & \begin{array}{c} 33.6677\\ 102.6748 \end{array} & \begin{array}{c} 80.8368\\ 29.9387 \end{array} \end{array}\right]$$

Figure 15 shows the track-to-track matching results of the two vessels. Both vessels travel downstream with the same heading and speed; however, there is a fixed calculation error in the position. 

Figure 16 shows the results of the trajectories of three vessels. Vessels No. 1 and 2 are running against the water, while vessel No. 3 is running along the water. The blue trajectory is the detected vessel trajectory in the image. In the large picture, the AIS trajectory length of the No. 1 vessel is larger than the trajectory detected by the image, because vessel No. 1 has a certain error in the detection result at the edge of the detected image. 

In Figure 17, the blue curve is the four vessel trajectories detected by the image, and the red curve indicates that only AIS trajectories of three vessels are available. The matching results show that vessel No. 1 has no corresponding AIS information.

## 5. Conclusions

This paper, focusing on maritime supervision, has proposed a multi-featured and multi-level matching algorithm for our Mar-UAV system. First, vessel feature information, extracted in aerial image and AIS information, was calibrated in time and space. Then, the feature matching was performed by the point matching and trajectory matching algorithm. Through the field experiments, it was proved that the proposed algorithm can solve the identification of vessels as well as illegal or dangerous acts. Such a matching algorithm combining aerial vision and AIS is beneficial to improve the autonomy of UAVs in the application of maritime supervision. The advantage of the algorithm is that more information about vessels could be recognized by combining vision and AIS. Through the proposed algorithm, some information (name, goods type, engine status, knot, etc.) of the detected vessel would be achieved from AIS. The information is helpful for maritime staffs to supervise the vessels properly. As one weakness, it seems unnecessary to employ all available information of vessels for matching. In the future, some machine learning method like Principal Component Analysis (PCA) will be used for feature selection from the information of vision and AIS.

## Figures and Tables

**Table 1:** The specific parameters of the employed drone. http://www.keweitai.com/products_detail/productId=38.html.

Types	Parameter
Maximum size of the whole machine	1710 ± 20 mm
Motor wheelbase	955 ± 10 mm
Standard takeoff weight	8.1 kg
Maximum takeoff weight	10.7 kg
Task load	≤3 kg
No-load hover time	≥50 min
Maximum wind resistance	Level 6 wind
Maximum flight speed	12 m/s
Maximum flight height	1000 m
GPS hover accuracy	Vertical direction: ±1.5 m Horizontal direction: ±2 m
Remote maximum control distance	7 km
Ground station maximum control distance	10 km

**Table 2:** The decoding results of the vessel’s dynamic information.

Description	Decoding Information
Type of information	1
Status	Engine in use
MMSI	413791052
Ground heading	227.9°
Ground speed	3.8 kn
Longitude	114.34549°
Latitude	30.6284433°

**Table 3:** The decoding results of the vessel’s static information.

Description	Decoding Information
Type of information	5
Name	HANGJUN14
MMSI	412070210
Type	Cargo ship
Distance from the reference point to the bow	48 m
Distance from the reference point to the stern	25 m
Distance from the reference point to left chord	12 m
Distance from the reference point to right chord	2 m
